# Dislocation of the Elbow; a New Method of Reduction

**Published:** 1869-11

**Authors:** Thomas Waterman

**Affiliations:** Boston


### Dislocation of the Elbow; a New Method of Reduction.

By Thomas Waterman, M. D.

[Re-printed from the Boston Medical and Surgical Journal, Vol. IV., Nos. 12-13, New Series.]

---

Finding no record in the surgical text-books of the method described below, I have thought the following case and comments worthy of publication.

On the 9th of May last, I was called to visit Mrs. L., æt. 30. She stated that, when near the bottom of a flight of stairs, she had tripped and fallen down the last three steps, striking with the whole weight of the body on her extended hand. As the accident had happened but half an hour previously, there was no swelling to mask the lesion. The left elbow was flexed at a right angle, and all motions were attended with great pain. After etherization, the ulna was found to be dislocated directly backwards at the elbow, as shown by the unusual prominence of the olecranon, depressions on either side of the triceps tendon, and resistance to complete extension of the forearm, which was twisted and pronated. The head of the radius rotated in its normal position, and no other lesion—neither dislocation nor fracture—could be detected.

Assuming that the patient's statement was correct, it seems strange, in view of the intimate connection of the carpal bones with the lower extremity of the radius, that Colle's fracture of that bone did not occur; or failing this, that the head of the radius was not forced out of place, either alone or in addition to the dislocation of the ulna.

Faithful trials of Sir Astley Cooper's method of bending the arm over the knee, and Mr. Skey's method of extending the forearm directly downwards in a line with the upper arm, failed to produce any effect.

I then succeeded in reducing the dislocation by bending the forearm backwards beyond a straight line, when, without any extension downwards the ulna returned to its normal position with a slight shock. An internal angular splint was applied, and evaporating lotions recommended. In eight days the splint was removed, the patient allowed to carry the arm in a sling and to execute slight motions in the joint daily.

The *modus operandi* of this method is as follows, viz:—when the ulna is dislocated backwards at the elbow without fracture of the coronoid process, the latter occupies the olecranon depression of the lower end of the humerus, and often requires considerable force to remove it from its abnormal position. By the method above described, the forearm is used as a lever, with the power (hand of the surgeon) at one end, the fulcrum (olecranon) at the other end, and the weight to be moved (coronoid process) between. As the forearm is extended backwards beyond a straight line, the olecranon impinges against the lower end of the humerus and becomes a fixed point or fulcrum; by continuing the forced extension, the coronoid process is lifted out of the olecranon depression of the humerus, and when this is accomplished, the tonic contraction of the brachialis anticus muscles restores the ulna to its natural place.

It will be seen that this method of reduction is exactly the reverse of the process by which the bone becomes dislocated, although it returns by the same path by which it escaped; these two facts, it seems to me, should be borne in mind in the reduction of all dislocations, and additional proof of this statement may be derived from a study of Prof. H. J. Bigelow's system of reducing dislocations of the hip by manipulation, and Dr. Crosby's method of reducing dislocations of the thumb.

The method is capable of the most decisive demonstration with macerated specimens of the ulna and humerus, and might be employed in dislocations of both radius and ulna backwards. It would be especially efficient in the reduction of old dislocations after the adhesions have been thoroughly broken up,

Since writing the above I have noticed in a late number of this JOURNAL the account of a case, copied from the London *Medical Times and Gazette* for July 17th, 1869, p. 79, in which essentially the same method, *i. e.* excessive extension, was successfully applied to the reduction of a vertical dislocation of the patella.

*Boston, October*, 1869.

